# Novel Herbal Therapeutic YH23537 Improves Clinical Parameters in Ligature-Induced Periodontal Disease Model in Beagle Dogs

**DOI:** 10.1155/2023/8130287

**Published:** 2023-04-29

**Authors:** Jang-Woo Shin, Eui-Ri Lee, Hyunwoo Noh, Jiyoon Kwak, Ji-Yeong Gal, Hyun-Je Park, Seongkyu Kim, Hyun-Kyung Song, Kangmoon Seo, Beom Seok Han

**Affiliations:** ^1^Yuhan R&D Institute, 25 Tapsil-ro, 35 Beon-gil, Giheung-gu, Yongin-si 446-902, Gyeonggi-do, Republic of Korea; ^2^Department of Veterinary Clinical Science, College of Veterinary Medicine, Seoul National University, 1 Gwanak-ro, Gwanak-gu, Seoul 151-742, Republic of Korea; ^3^Yuhan Natural Product R&D Center, Yuhan Care Co., Ltd., 25 Tapsil-ro, 35 Beon-gil, Giheung-gu, Yongin-si, Gyeonggi-do, Republic of Korea; ^4^Department of Bio Applied Toxicology, Toxicology Research Center, Hoseo University, 20 Hoseo-ro, 79 Beon-gil, Baebang-eup, Asan-si, Chungcheongnam–do, Republic of Korea

## Abstract

Currently, available medicine does not satisfy the clinical unmet needs of periodontal disease. Therefore, novel drugs with improved efficacy profiles are needed. We previously demonstrated that YH14642, water extracts of Notoginseng Radix and Rehmanniae Radix Preparata, improved probing depths in double-blind phase II clinical trial. However, it still has hurdles for commercialization due to the low efficiency of active compound extraction. To resolve this issue, we developed YH23537 through process optimization to extract active compounds efficiently while still achieving the chemical profile of YH14642. In this study, we investigated the therapeutic effects of YH23537 compared with YH14642 using a canine model of ligature-induced periodontitis. Human gingival fibroblast (hGF) cells were treated with various concentrations of YH23537 or YH14642 with lipopolysaccharide (LPS) for 24 hr. IL-6 and IL-8 levels in the conditioned media were determined using Luminex. Sixteen 3-year-old male beagle dogs had their teeth scaled and polished using a piezo-type ultrasonic scaler under general anesthesia and brushed once daily for the following 2 weeks. Two weeks after the scaling procedure, the left upper second premolar (PM2), third premolar (PM3), and fourth premolar (PM4) as well as the left lower PM3, PM4, and first molar (M1) were ligated with silk-wire twisted ligatures. The dogs were fed with soft moistened food to induce periodontitis for 8 weeks, and the ligatures were then removed. YH23537 and YH14642 were administered for 4 weeks, and clinical periodontal parameters such as plaque index (PI), gingival index (GI), probing depth (PD), clinical attachment level (CAL), and bleeding on probing (BoP) were determined before and 1, 2, 3, and 4 weeks after treatment. YH23537 inhibited IL-6 and IL-8 secretion in a dose-dependent manner in hGF cells stimulated with LPS. The IC_50_ values for YH23537 were 43 and 54 *μ*g/ml for IL-6 and IL-8, respectively, while the values for YH14642 were 104 and 117 *μ*g/ml, respectively. In the animal study, clinical parameters including GI, PD, CAL, and BoP were significantly increased after 8 weeks of ligature-induced periodontitis. The YH23537 300 and YH23537 900 mg groups had significant improvements in CAL from 1 to 4 weeks after treatment in comparison to the placebo group. GR values in the YH23537 900 mg group were decreased throughout the treatment period. GI values were also reduced significantly after 4-week treatment with 300 and 900 mg of YH23537. YH23537 at 300 mg doses showed comparable efficacy for CAL and GR with 1,000 mg of YH14642. YH23537 showed therapeutic efficacy against periodontitis in dogs, mediated by anti-inflammatory effects. These findings indicate that YH23537 has the potential for further development as a new drug for patients suffering from periodontal disease.

## 1. Introduction

Periodontal disease is a bacterial biofilm-induced chronic inflammatory disease that occurs in tooth-supporting tissues such as gingiva, periodontal ligament, cementum, and bone [1], which are highly prevalent, with an estimated 90% of the population affected worldwide [1]. In the United States, the prevalence of periodontal diseases in adults has significantly increased by age and smoking status, reporting ∼42.2% with 7.8% of severe disease cases [2]. The initial stage of periodontal disease is gingivitis caused by the bacteria in accumulated dental plaque. The bacteria trigger an inflammatory response in the gum tissues, leading to redness, swelling, and bleeding. Untreated gingivitis can progress to periodontitis, which is characterized by deeper pockets, bone loss, and tooth mobility. The pathogenesis of periodontal disease involves a complex interplay between bacterial plaque, host immune response, and genetic and environmental factors. Periodontal disease is also associated with systemic diseases including atherosclerosis, diabetes, inflammatory bowel disease, Alzheimer's disease, and possibly rheumatoid arthritis [3–7]. Recent studies have shown that miRNAs can serve as biomarkers, and targeting specific miRNAs has also been proposed as a therapeutic strategy for periodontitis [8]. Circulating cells, such as monocytes and neutrophils, play crucial roles in the pathogenesis of periodontitis by migrating from the bloodstream to the periodontal tissues and contributing to inflammation and tissue destruction [9].

Current therapeutic strategies for periodontal disease are dominated by dental procedures and techniques such as scaling and root planing (SRP) and surgery, with antibiotics used where necessary. However, due to limitations, some studies have tried to improve therapeutic effects by using locally applied antibiotics (LAAs) or oral antibiotics (low-dose doxycycline). Currently-available products including Arestin®, Periochip®, Atridox®, and Periostat® have been launched as a result of these efforts. However, due to the significant unmet needs that remain for periodontal disease, novel drugs with improved efficacy profiles are needed.

Previously, we demonstrated that an earlier candidate, YH14642, improved probing depths (“A Double-blind, Randomized, Parallel, Placebo-active Controlled, Multi-center Phase II Clinical Trial to Investigate the Efficacy and Safety of YH14642 Following 12-week Oral Administration in Patients with Chronic Periodontal Disease (NCT01499225)”). YH14642 has shown anti-inflammatory effects in human monocytic cells and hGF and can reduce alveolar bone loss and MMP-9 expression in rats with LPS-induced periodontitis [10]. However, as the extraction rate of ginsenosides from Notoginseng Radix was previously low, we subsequently developed YH23537 through optimization of the YH14642 manufacturing process. YH23537 reduced the severity of pain and articular cartilage degradation in monosodium iodoacetate- (MIA-) induced osteoarthritis (OA) rats and oxidative injury, inflammatory mediators, and inducing anabolic factors were suppressed in OA joint by YH23537 treatment [11]. In this study, we compared the therapeutic efficacy between YH23537 and YH14642 in a canine model of periodontitis.

## 2. Materials and Methods

### 2.1. Preparation of YH23537 and YH14642

Notoginseng Radix and Rehmanniae Radix Preparata were identified using the Korean Herbal Pharmacopoeia and the Korean Pharmacopoeia. YH14642 was manufactured according to bulk good manufacturing practices by Sungilbioex (Gyeonggi-do, Korea). Briefly, a mixture of 130 kg of Notoginseng Radix and 14.4 kg of Rehmanniae Radix Preparata (9 : 1) were boiled in 1,870 L of water for 8 hr. The extracted solution was filtered, concentrated, and then spray-dried. YH23537 was manufactured according to bulk good manufacturing practices by Bolak (Gyeonggi-do, Korea). Briefly, a mixture of 120 kg of Notoginseng Radix and 15 kg of Rehmanniae Radix Preparata (8 : 1) was extracted twice in 945 and 675 L of 50% ethanol for 4 and 2 hr, respectively. The extracted solution was filtered and concentrated to remove the ethanol. The resultant solution was boiled for 10 hr, filtered, and mixed with dextrin at a 1 : 1 ratio of solid content before spray drying.

YH14642 and YH23537 were tested for the presence of heavy metals (total heavy metal ≤ 30 ppm; AS ≤ 3 ppm; and Pb ≤ 5 ppm), pesticide residues (BHC (sum of *α*, *β*, *γ* and *δ*-BHC) ≤0.2 ppm, DDT (sum of *o*,*p*-DDE, *p*,*p*-DDE, *o*,*p*-DDT, *p*,*p*-DDT) ≤0.1 ppm, aldrin + dieldrin + endrin ≤ 0.01 ppm), general bacteria (≤1 × 10^5^ CFU/g), fungi (≤100 CFU/g), and specific pathogens (negative for *Escherichia coli*, *Pseudomonas aeruginosa*, *Staphylococcus aureus*, and *Salmonella* spp.), and were also subjected to quantitation tests (G. Rb1 ≥ 9.0 mg/g in extract).

YH14642 tablet (500 mg) was manufactured according to good manufacturing practice in factory of Yuhan Corporation (Chungcheongbuk-do, Korea), and YH23537 tablets (37.5, 150, 450 mg) were prepared in R&D Institute of Yuhan Corporation (Gyeonggi-do, Korea). Placebo tablet was prepared with the same size and coating tablets by replacing the active drug ingredient with an excipient.

### 2.2. Composition of YH23537 and High-Performance Liquid Chromatography-Based Fingerprinting

Five hundred grams of YH23537 or YH14642 were placed into 50 ml volume flask, and then 40 ml of 50% methanol was added into the flask. After sonication for 1 hr, 50% methanol was added up to 50 ml, and the prepared sample was filtered by 0.45 *µ*m of regenerated cellulose filter. For identifying the profile of ginsenosides, HPLC method was performed using a Waters Alliance LC (Waters Corporation, MA, USA) equipped with a quaternary gradient pump and a sample manager. An HPLC method was developed using RP column (Zorbax SB C18, 4.6 × 150 mm, 3.5 *µ*m; Agilent, CA, USA). The column was maintained at 30°C. A 20 *µ*l aliquot was injected onto the column. The gradient elution system consisted of 5% acetonitrile (A) and 95% acetonitrile (B) and separation was achieved using the following gradient: 0–3 min, 5% B; 3–18 min, 5%–50% B; 18–28 min, 50%–90% B; 28–40 min, 90% B; 40–41 min, 90%–5% B; 41–45 min, 5% B. The flow rate was 1.0 ml/min and the eluate was monitored at 203 nm by an ultraviolet detector.

### 2.3. Anti-Inflammatory Effects *In Vitro*

Human gingival fibroblasts (hGF) were obtained from Creative Bioarray (NY, USA) and cultured with SuperCult® Fibroblast Medium (Creative Bioarray, NY, USA) at 37°C in 5% CO_2_. hGF cells were seeded into 96-well plates (1 × 10^4^ cells /well) and cultured for 24 hr. The cells were treated with various concentrations of YH23537 or YH14642 with 1 *μ*g/ml of *Porphyromonas gingivalis* LPS (InvivoGen, CA, USA) for 24 hr. The conditioned media was harvested and stored at −70°C until analysis. Cell viability was determined by WST assay (Daeillab Service, Seoul, Korea). The concentrations of IL-6 and IL-8 in the media were measured using a Magnetic Luminex® Screening Assay kit (R&D Systems, Minneapolis, USA) according to the manufacturer's protocol.

### 2.4. Animals and Experimental Design

Sixteen 3-year-old male beagle dogs (Marshall, USA) were fed with commercial pellets (2025C) (Harlan, IN, USA) and tap water *ad libitum* before the experiment began. All teeth were scaled and polished using a piezo-type ultrasonic scaler (BonArt, LA, USA) under general anesthesia induced by a combination of medetomidine (0.01 mg/kg; Orion Pharma, Finland), tramadol (2 mg/kg; Samsung Pharm, Korea), zolazepam and tiletamine (2.5 mg/kg; Virbac, France) administered *via* intramuscular injection, and the teeth were brushed once daily for the following 2 weeks. Two weeks after scaling, all animals except one animal in the sham control group had ligation with silk-wire twisted ligatures (SWL) according to the described method by Kim et al. [12] on the left upper second premolar (PM2), third premolar (PM3), and fourth premolar (PM4) as well as the left lower PM3, PM4, and first molar (M1). The dogs were fed with soft moistened food to induce periodontitis for 8 weeks. After 8-week induction, clinical periodontal parameters were assessed and the dogs with SWL removed were assigned to five groups (Placebo, YH23537 75, YH23537 300, YH23537 900, YH14642 1,000 mg; three animals per group) based on CAL values. Placebo tablets, YH23537 tablets (37.5, 150, 450 mg), or YH14642 tablets (500 mg) were administrated orally twice a day.

The clinical periodontal parameters were recorded to evaluate the periodontal status before ligation, before treatment, and at 1, 2, 3, 4 weeks after treatment. These parameters included plaque index (PI), gingival index (GI), probing depth (PD), clinical attachment level (CAL), and bleeding on probing (BoP).

The PI was rated as follows: PI 0: no plaque, PI 1: a film of plaque adhering to the free gingival margin and adjacent area of the tooth (not more than 1 mm), PI 2: moderate accumulation of soft deposits within the gingival pocket, or on the tooth and gingival margin (less than one half of crown), PI 3: abundance of soft matter within the gingival pocket and/or on the tooth and gingival margin (more than one half of the crown). Gingival index (GI) was rated as follows: (a) GI 0: absence of gingival inflammation, (b) GI 1: mild gingival inflammation—slight change in color of gingival margin and little change in texture, (c) GI 2: Moderate gingival inflammation—moderate glazing, redness, edema, hypertrophy, and/or bleeding on pressure, (d) GI 3: severe gingival inflammation, marked redness and hypertrophy, spontaneous bleeding and ulceration. Probing depth (PD) was defined as the distance between the gingival margin and the bottom of the probable pocket; clinical attachment level (CAL): the distance between the cement–enamel junction and the bottom of the probable pocket. CAL and PD were measured at three sites per tooth. No ligature casts were dislodged during the experiment. All measurements were taken by an experienced clinician (E.R. Lee, Seoul National University, Korea) using a Williams periodontal probe (Osung MND, Korea). Gingival recession was calculated using PD and CAL. Bleeding on probing (BoP) was assessed as probing (a) which was followed by no bleeding, referred to BoP 0 and (b) with bleeding, referred to as BoP 1.

The experiments were conducted in accordance with the rules for Use of Laboratory Animals as adopted and promulgated by the U.S. National Institutes of Health following approval of the Institutional Animal Care and Use Committee of Yuhan Corporation (Authorization number, 14172). The animal housing was controlled at 23 ± 2°C with 50 ± 10% relative humidity under a 12/12 hr light/dark cycle throughout the experimental period.

### 2.5. Statistical Analysis

Chemical profiles were analyzed using Pearson correlation analysis. Data are shown as the mean ± standard error (SEM). Statistical analysis of the data was carried out using one-way ANOVA and Tukey's multiple-comparison test or Dunnett's multiple comparison test, with values of *P* < 0.05 considered to be statistically significant.

## 3. Results

### 3.1. YH23537 Has a Similar Chemical Profile to YH14642

**Figure 1:**
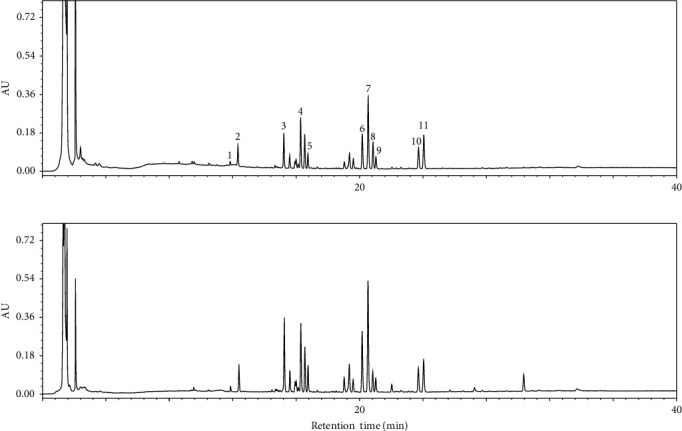
Chemical fingerprint chromatograms of YH14642 (a) and YH23537 (b). Notoginsenoside R1 (1), ginsenoside Rg1 (2), Rb1 (3), 20(S)-Rh1 (4), Rd (5), Rh4 (6), Rk3 (7), 20(S)-Rg3 (8), 20(R)-Rg3 (9), Rg5 (10), Rk1 (11).

The peaks were identified by comparing the retention times of the peaks with those of the reference compounds eluted under the same conditions. The investigated saponins were well separated, and the comparison of chemical profile between YH14642 and YH23537 was performed (Figure 1). Pearson correlation coefficient was analyzed using 20 peak areas (Table S1), and *R*-value was 0.947 (*P* < 0.0001).

### 3.2. YH23537 Inhibits Inflammatory Cytokines in Pg-LPS-Stimulated Gingival Fibroblasts

**Figure 2:**
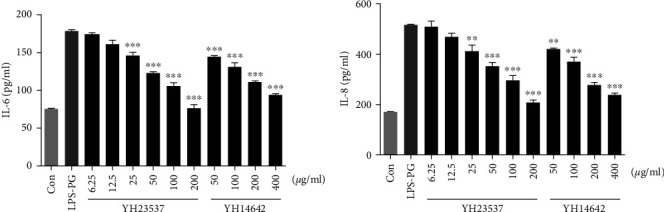
IL-6 and IL-8 production in hGF cells stimulated with LPS-PG. Human gingival fibroblast cells (1 × 10^4^) were seeded into 96-well plates. The cells were treated with YH14642 or YH23537 with *Porphyromonas gingivalis* LPS (LPS-PG, 1 *μ*g/ml) for 24 hr. IL-6 (a) and IL-8 (b) levels in conditioned media were determined using Luminex. Data represent the mean ± SD.  ^*∗∗*^*P* < 0.01 and  ^*∗∗∗*^*P* < 0.001 compared with the LPS-PG.

We first investigated the anti-inflammatory effects of YH23537 *in vitro*. After stimulation of hGF cells with LPS from *P. gingivalis*, IL-6 production increased to 179 *μ*g/ml compared with 76 *μ*g/ml present in the control cells. When YH23537 was co-treated simultaneously, IL-6 secretion was reduced in a dose-dependent manner. Similarly, YH23537 also significantly inhibited IL-8 secretion dose-dependently. The YH23537 IC_50_ values for IL-6 and IL-8 were 43 and 54 *μ*g/ml, while the YH14642 IC_50_ values for IL-6 and IL-8 were 104 and 117 *μ*g/ml, respectively (Figure 2).

### 3.3. SWL-Induces Periodontal Disease in Dogs

Animals were randomized based on CAL value postperiodontal disease induction by silk-wire twisted ligatures for 8 weeks. Initial clinical parameters PI, GI, PD, CAL, GR, and BoP were in a range of between 1.39 and 2.28, 2.11 and 2.39, 2.55 and 2.81, 2.69 and 2.82, 0.00 and 0.26, 0.33 and 0.70, respectively, in induced groups whereas 0.83, 0.00, 1.81, 0.00, −1.81, and 0.22, respectively, in the sham control group (Table 1).

### 3.4. YH23537 Improves Clinical Symptoms in Periodontitis Dogs

**Figure 3:**
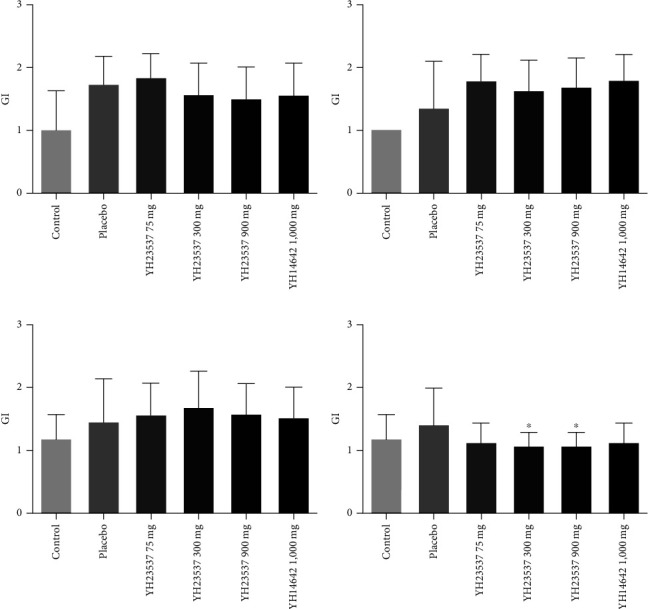
GI in ligature-induced periodontitis dogs. The left upper second premolar (PM2), third premolar (PM3), and fourth premolar (PM4) as well as the left lower PM3, PM4, and first molar (M1) were ligated with silk-wire twisted ligatures and the dogs were fed with soft moistened food. After 8 weeks, the ligatures were removed and the dogs were administered with YH23537 or YH14642 for 4 weeks. CAL was measured every week throughout the experimental period ((a) week 1; (b) week 2; (c) week 3; (d) week 4). Data represent the mean ± SD.  ^*∗*^*P* < 0.05 compared with the placebo group.

**Figure 4:**
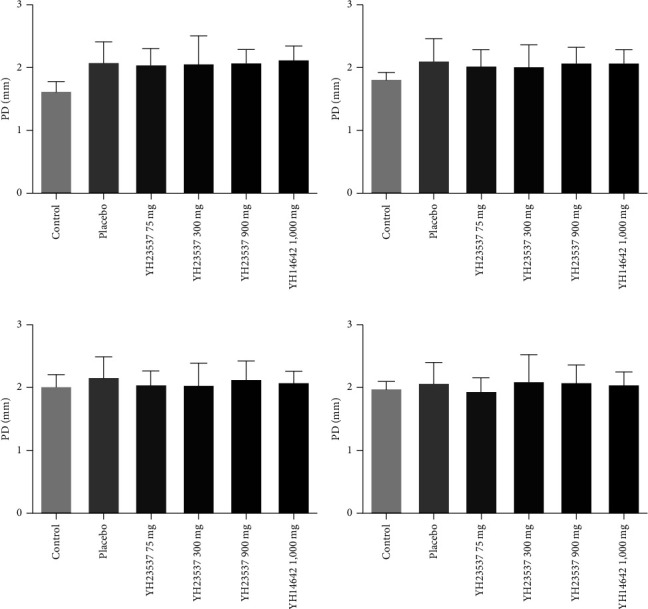
PD in ligature-induced periodontitis dogs. The left upper second premolar (PM2), third premolar (PM3), and fourth premolar (PM4) as well as the left lower PM3, PM4, and first molar (M1) were ligated with silk-wire twisted ligatures and the dogs were fed with soft moistened food. After 8 weeks, the ligatures were removed and the dogs were administered with YH23537 or YH14642 for 4 weeks. CAL was measured every week throughout the experimental period ((a) week 1; (b) week 2; (c) week 3; (d) week 4). Data represent the mean ± SD.

**Figure 5:**
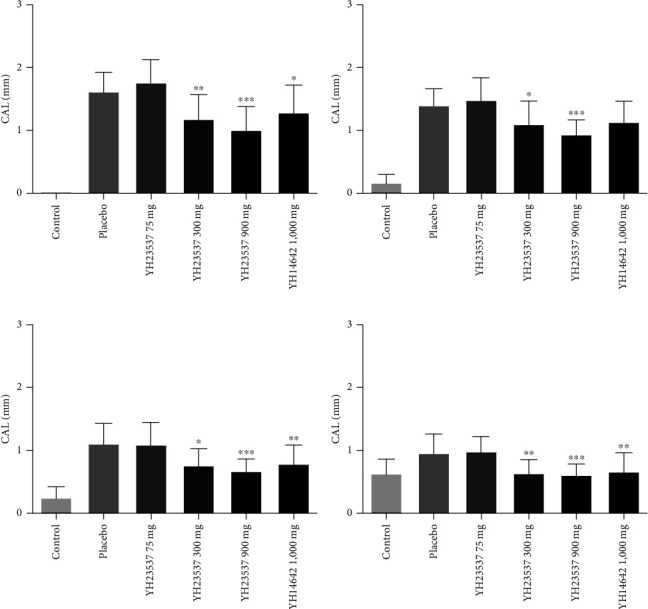
Clinical attachment level (CAL) in ligature-induced periodontitis dogs. The left upper second premolar (PM2), third premolar (PM3), and fourth premolar (PM4) as well as the left lower PM3, PM4, and first molar (M1) were ligated with silk-wire twisted ligatures and the dogs were fed with soft moistened food. After 8 weeks, the ligatures were removed and the dogs were administered with YH23537 or YH14642 for 4 weeks. CAL was measured at every week throughout the experimental period ((a) week 1; (b) week 2; (c) week 3; (d) week 4). Data represent the mean ± SD.  ^*∗*^*P* < 0.05,  ^*∗∗*^*P* < 0.01, and  ^*∗∗∗*^*P* < 0.001 compared with the placebo group.

We measured clinical parameters every week before and after treatment with YH23537. PI values did not show difference between the groups during the experimental period. GI did not improve following YH23537 treatment until 3 weeks but was decreased significantly compared with the placebo group in YH23537 300 and 900 mg-treated dogs after 4 weeks of treatment (Figure 3). PD was recovered within 2 weeks. From 2 to 4 weeks, the PD values for all control groups were similar (Figure 4). One week after drug treatment, CAL was significantly improved in a dose-dependent manner at 1.60, 1.74, 1.17 mm (*P* < 0.01), and 1.00 mm (*P* < 0.001) in the Placebo, YH23537 75, YH23537 300, and YH23537 900 mg groups, respectively, after 1-week treatment, while YH14642 reduced CAL by 1.27 mm (*P* < 0.05) at the same time. By the 2nd week, CAL improvement was sustained in dogs administered with 300 mg (*P* < 0.05) and 900 mg (*P* < 0.001) of YH23537. YH23537 300 and 600 mg and YH14642 1,000 mg doses improved CAL values significantly from the 3rd to 4th week (Figure 5).

At each point, the CAL values were 1.27, 1.11, 0.76, and 0.65 mm in the YH14642 1,000 mg group while the CAL values were 1.17, 1.07, 0.74, and 0.61 mm in the YH23537 300 mg group. Therefore, the efficacy of the YH23537 300 mg dose was comparable or superior to YH14642 1,000 mg.

**Figure 6:**
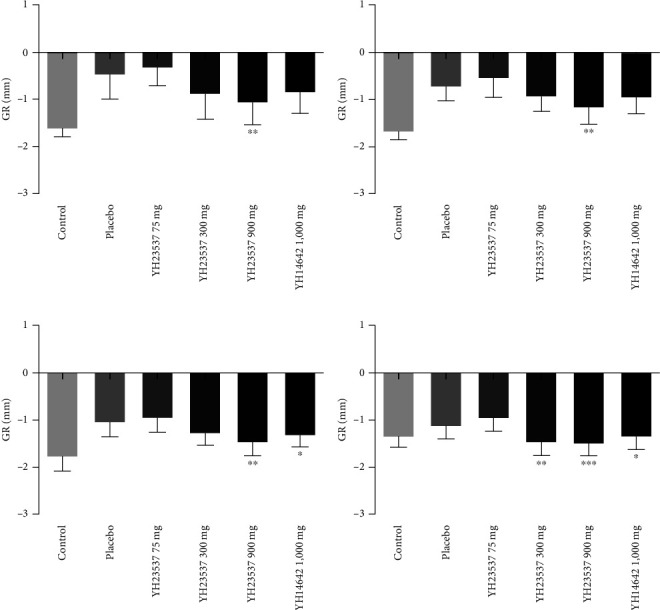
GR in ligature-induced periodontitis dogs. The left upper second premolar (PM2), third premolar (PM3), and fourth premolar (PM4) as well as the left lower PM3, PM4, and first molar (M1) were ligated with silk-wire twisted ligatures and the dogs were fed with soft moistened food. After 8 weeks, the ligatures were removed and the dogs were administered with YH23537 or YH14642 for 4 weeks. CAL was measured every week throughout the experimental period ((a) week 1; (b) week 2; (c) week 3; (d) week 4). Data represent the mean ± SD.  ^*∗*^*P* < 0.05,  ^*∗∗*^*P* < 0.01, and  ^*∗∗∗*^*P* < 0.001 compared with the placebo group.

GR was calculated using the PD and CAL values. YH23537 treatment reduced GR values at −0.30, −0.88, and −1.06 mm compared with −0.48 mm in the placebo group. GR was continuously reduced until the 4th week, with values for YH23537 300 and 600 mg and YH14642 1,000 mg-treated dogs at −1.47, −1.49, and −1.38 mm, respectively, and significantly different from that of the placebo at −1.13 mm (Figure 6). YH14642 1,000 mg elicited a very similar change in GR compared with the YH23537 300 mg dose throughout the treatment period.

## 4. Discussion

We previously demonstrated that YH14642 improves periodontitis in patients with chronic periodontal disease by demonstrating significant improvements in probing depth. However, some ginsenosides present in Notoginseng Radix cannot be efficiently extracted with water. We found that by replacing water with 50% ethanol to extract Notoginseng power, the extraction rate was improved by almost 100%. Ginsenosides are active compounds present in ginseng species with pharmacological activities demonstrated in various disease models including arthritis, atherogenesis, ischemia–reperfusion brain injury, liver injury, colitis, and osteoporosis [13–18].

The prevalence of periodontitis in dogs is high and is known to increase with aging. Etiopathologically, it closely resembles the human disease [19, 20]. A number of studies on periodontitis have been conducted in dogs, and beagles are commonly used due to the size of their teeth and the similarity of periodontal tissue with humans [21]. In this study, SWLs were used to prevent loss of ligature during the experimental period [12]. After 8 weeks of ligature and a diet of soft food, periodontitis was inducted as confirmed by clinical markers including GI, CAL, PD, and BOP.

YH23537 administration significantly improved CAL values in a dose-dependent manner throughout the experimental period. GI was also alleviated after a 4-week treatment with YH23537. The therapeutic effects of YH23537 were approximately three-fold more potent than YH14642, with *in vivo* data consistent with the *in vitro* data.

The immune-inflammatory response develops in the gingival and periodontal tissues in response to sustained bacteria challenge. During this inflammatory process, proteolytic enzymes and chemotactic factors are released, which destroy tissues and recruit leucocytes to the inflamed area. Plaque and bleeding on probing are related to clinical attachment loss [22]. IL-8 is one of the most abundantly expressed chemokines in the oral cavity [23] and is produced by monocytes, macrophages, fibroblasts, keratinocytes, and endothelial cells and is responsible for the recruitment and activation of neutrophils. IL-8 levels in the gingival crevicular fluid (GCF) of patients with aggressive periodontitis have been shown to be significantly higher than those with chronic periodontitis, gingivitis, and periodontally healthy groups, which correlate well with clinical parameters including BOP, PI, GI, PD, and CAL [24]. IL-6 is an inflammatory cytokine that has a positive correlation with bleeding and PD [25]. YH23537 inhibited the production of IL-6 and IL-8 in human gingival fibroblasts stimulated with PG-LPS, a finding that was expected given that *Panax notoginseng* inhibits MMP-2 expression in periodontal ligament fibroblasts and attenuates osteoclastogenesis LPS-induced proinflammatory mediators in RAW264.7 cells [26, 27]. The anti-inflammatory effects of individual ginsenosides have also been previously investigated. Rb1 is a major component of YH23537 and has been reported to reduce inflammatory cytokines TNF-*α*, IL-6, and IL-1*β* released in LPS-stimulated RAW264.7 cells and bone-marrow-derived macrophages, exhibiting anti-inflammatory effects *in vitro* and in disease animal models such as collagen-induced arthritic mice [28–30]. A further component, Rd, exhibited both inhibitory activity for iNOS and COX-2 expression in LPS-stimulated RAW264.7 cells as well as suppressive activity in carageenan-induced inflammation and periodontitis by ligaturing sterile sutures [31–33]. Rg3 also suppressed proinflammatory cytokine production, such as TNF-*α*, IL-1*β*, and IL-6 in RAW264.7 cells [34]. Zhang et al. [35] reported recently that Rg1, Rg3, Rg5, Rb1, and Rh2 all improved arthritis index and joint histopathologic score and reduced TNF-*α* and IL-6 expression in the joints in collagen-induced arthritis mouse model. YH23537 improved joint histopathologic scores and reduced IL-1*β* and IL-6 expression in OA joints in MIA-induced arthritis rats [11]. Therefore, the anti-inflammatory effects of YH23537 are likely to be a primary factor in its therapeutic effects on periodontitis.

Nonsteroidal anti-inflammatory drugs (NSAIDs), such as ibuprofen and aspirin, can help reduce inflammation and relieve pain associated with periodontal disease. But systemic literature review demonstrated that adjuvant NSAID drugs with SRP did not improve PD and CAL [36]. In general, the most effective approach in managing periodontal disease involves a combination of strategies, including proper oral hygiene practices, professional cleanings, antibiotic therapy, and, in some cases, surgical or advanced nonsurgical interventions due to the complex interplay between bacterial plaque, host immune response, and genetic and environmental factors in the pathogenesis of periodontal disease. So, the anti-inflammatory effects alone may not be sufficient to address all of the efficacy of YH23537 in this model. YH23537 has various biologically known or unknown active compounds which result in anti-inflammatory, antimicrobial, tissue-regenerating, and antioxidative stress effects [27, 30, 31, 33, 37, 38]. For example, ginsenoside Rg1 was found to stimulate the proliferation and differentiation of human periodontal ligament cells [39] while grape seed extracts, green tea extracts, and horse chestnut leaf extracts, containing various active compounds, have demonstrated to alleviate experimental periodontitis [40–42]. Furthermore, other active compounds, such as catechin, epigallocatechin-3 gallate, curcumin, oleuropein, and baicalin, also have proved antiperiodontitis effects in animal models [43–46].

YH23537, with its various beneficial effects, could potentially be a useful therapeutic agent for the management of periodontal disease, but further research is still needed to fully understand its efficacy and mechanisms of the action.

## 5. Conclusion

Taken together, our results demonstrate that YH23537 has therapeutic efficacy for periodontitis, which is likely due to its documented anti-inflammatory properties. Further clinical studies are needed to more comprehensively assess the suitability of this novel therapeutic for wider use.

## Figures and Tables

**Table 1:** Baseline of clinical parameters after randomization.

Parameters	Control	Placebo	YH23537 75 mg	YH23537 300 mg	YH23537 900 mg	YH14642 1,000 mg
PI	0.83 ± 0.41	1.39 ± 0.85	2.28 ± 0.67^*∗∗*##^	1.50 ± 0.86	2.06 ± 0.80^*∗*^	1.50 ± 0.71
GI	0.00 ± 0.00	2.28 ± 0.57	2.11 ± 0.32	2.33 ± 0.59	2.39 ± 0.70	2.17 ± 0.51
PD (mm)	1.81 ± 0.13	2.81 ± 0.41	2.58 ± 0.52	2.55 ± 0.23	2.57 ± 0.21	2.56 ± 0.23
CAL (mm)	0.00 ± 0.00	2.81 ± 0.56^*∗∗∗*^	2.70 ± 0.61^*∗∗∗*^	2.69 ± 0.40^*∗∗∗*^	2.78 ± 0.39^*∗∗∗*^	2.82 ± 0.32^*∗∗∗*^
GR (mm)	−1.81 ± 0.13	0.00 ± 0.35^*∗∗∗*^	0.12 ± 0.24^*∗∗∗*^	0.15 ± 0.27^*∗∗∗*^	0.21 ± 0.30^*∗∗∗*^	0.26 ± 0.24^*∗∗∗*^
BoP	0.22 ± 0.17	0.67 ± 0.26^*∗∗*^	0.70 ± 0.23^*∗∗*^	0.63 ± 0.19^*∗*^	0.61 ± 0.29^*∗*^	0.33 ± 0.34

Data represent the mean ± SD.  ^*∗*^*P* < 0.05,  ^*∗∗*^*P* < 0.01, and  ^*∗∗∗*^*P* < 0.001 compared with the control group.  ^##^*P* < 0.01 compared with the placebo group.

## Data Availability

The data used in this study are available upon reasonable request from the corresponding author.

## Supplementary Materials

Supplementary Materials Table S1: Comparison of chemical profile between YH14642 and YH23537.
