# Supplementary material for: Novel Herbal Therapeutic YH23537 Improves Clinical Parameters in Ligature-Induced Periodontal Disease Model in Beagle Dogs
Source: Int J Dent. 2023 Apr 29;2023:8130287. doi: 10.1155/2023/8130287 (PMC10163972; doi:10.1155/2023/8130287)
Supplement: Supplementary Materials — Table S1: Comparison of chemical profile between YH14642 and YH23537. [file 8130287.f1.docx]

**Title: N**ovel herbal therapeutic YH23537 improves clinical parameters in ligature-induced periodontal disease model in Beagle dogs

Jang-Woo Shin, Eui-Ri Lee, Hyunwoo Noh, Jiyoon Kwak,

Ji-Yeong Gal, Hyun-Je Park, Seongkyu Kim, Hyun-Kyung Song, Kangmoon Seo, Beom Seok Han

Corresponding author: Jang-Woo Shin, Ph D.

E-mail address: jwshin@yuhan.co.kr

| Peak | RT | RRT | Average Peak Area | |
| --- | --- | --- | --- | --- |
|  |  |  | **YH14642** | **YH23537** |
| Notoginsenoside R1 | 11.842 | 0.78 | 60203 | 72695 |
| Ginsenoside Rg1 | 12.332 | 0.81 | 355507 | 421495 |
| Ginsenoside Rb1 | 15.226 | 1.00 | 605253 | 1236933 |
| Unknown 1 | 15.591 | 1.02 | 223060 | 355733 |
| Unknown 2 | 15.924 | 1.05 | 107002 | 172689 |
| Unknown 3 | 15.998 | 1.05 | 164368 | 181644 |
| Unknown 4 | 16.125 | 1.06 | 87119 | 89263 |
| Ginsenoside Rh1 (20S) | 16.287 | 1.07 | 944829 | 1236236 |
| Unknown 5 | 16.543 | 1.09 | 633127 | 794512 |
| Ginsenoside Rd | 16.745 | 1.10 | 252296 | 442440 |
| Unknown 6 | 19.04 | 1.25 | 150577 | 295113 |
| Unknown 7 | 19.288 | 1.27 | 38769 | 60453 |
| Unknown 8 | 19.364 | 1.27 | 347247 | 600474 |
| Unknown 9 | 19.607 | 1.29 | 197408 | 237274 |
| Ginsenoside Rh4 | 20.173 | 1.32 | 768161 | 1272633 |
| Ginsenoside Rk3 | 20.543 | 1.35 | 1559826 | 2318732 |
| Ginsenoside Rg3 (20S) | 20.85 | 1.37 | 587529 | 424141 |
| Ginsenoside Rg3 (20R) | 21.036 | 1.38 | 250927 | 281541 |
| Ginsenoside Rk1 | 23.721 | 1.56 | 529934 | 571984 |
| Ginsenoside Rg5 | 24.054 | 1.58 | 849820 | 782489 |
| Pearson correlation |  |  |  | **0.947** |

Supplement Table 1. Comparison of chemical profile between YH14642 and YH23537
